# Is frequency of potato and white rice consumption associated with cardiometabolic risk factors in children and adolescents: the CASPIAN-V study

**DOI:** 10.1186/s12872-020-01524-y

**Published:** 2020-05-19

**Authors:** Fereshteh Baygi, Mostafa Qorbani, Mohammad Esmaeil Motlagh, Gita Shafiee, Kourosh Nouri, Zeinab Ahadi, Armita Mahdavi-Gorab, Ramin Heshmat, Roya Kelishadi

**Affiliations:** 1 Center of Maritime Health and Society, Department of Public Health, University of Southern Denmark, Esbjerg, Denmark; 2 Non-communicable Diseases Research Center, School of Medicine, Alborz University of Medical Sciences, Baghestan Blvd, Karaj, 31485/56 Iran; 3 Chronic Diseases Research Center, Endocrinology and Metabolism Population Sciences Institute, Tehran University of Medical Sciences, Tehran, Iran; 4 Department of Pediatrics, Ahvaz Jundishapur University of Medical Sciences, Ahvaz, Iran; 5 Student Research Committee, Alborz University of Medical Sciences, Karaj, Iran; 6 Child Growth and Development Research Center, Research Institute for Primordial Prevention of Non-communicable Disease, Isfahan University of Medical Sciences, Isfahan, Iran

**Keywords:** Cardiometabolic risk factors, Metabolic syndrome, White Rice, Bread, Potato

## Abstract

**Background:**

This study evaluated the association of frequency of potato and rice consumption with cardiometabolic risk factors in children and adolescents.

**Methods:**

This nationwide cross-sectional survey was conducted on 14,400 children and adolescents. Fasting blood was obtained from a sub-sample of 4200 randomly selected students. Physical examination and laboratory tests were conducted under standard protocols. Metabolic Syndrome (Mets) was defined based on the Adult Treatment Panel III criteria modified for the pediatric age group. The self-reported frequency consumption of white rice and potato was reported on a daily or non-daily basis.

**Results:**

The participation rate for the whole study and for blood sampling were 99 and 91.5%, respectively. Overall, 49.4% of the participants were girls while 50.6% were boys. The frequency of daily consumption of white rice and potato was 84.4 and 21.3%, respectively. In the multivariable linear regression model, daily consumption of potato increased body mass index (β: 0.05, SE: 0.20, *p* = 0.010), waist (β: 0.63, SE: 0.24, *p* = 0.008), and hip circumferences (β: 0.62, SE: 0.26, *p* = 0.019). Moreover, in the multivariable logistic regression, daily consumption of potato was significantly associated with an increased risk of overweight (OR: 1.21, 95% CI: 1.04–1.39, *P* = 0.012). The potato and rice consumption had no statistically significant association with other cardiometabolic risk factors.

**Conclusions:**

Daily consumption of potato was significantly associated with higher anthropometric measures, whereas rice consumption had no statistically significant association with cardiometabolic risk factors. Future research to examine the possible obesogenic effects of intake of potato on children and adolescents is recommended.

## Background

Nutrition -related non-communicable diseases like overweight and obesity are increasing in Iran [1–3]. The prevalence of overweight and obesity in children of America, Europe, Western Pacific, and Southeast Asia is 31, 38, 27, and 22%, respectively [4]. The development of cardiovascular diseases- as one of the main causes of death- is related to numerous early life risk factors such as overweight and obesity [5, 6]. Childhood excess weight increases the risk of obesity or overweight in the later years [7]. Shifts in dietary pattern can have significant effects on weight changes in terms of excess weight in children and adolescents [8, 9]. Longitudinal studies have shown that there is an association between dietary patterns and cardiovascular diseases [10, 11]. Numerous studies have been published showing an inverse association between the consumption of whole-grain foods and body weight so that consumption of whole grains can reduce hunger and increase satiety [12, 13]. Based on the results of a national household consumption survey, major part of the Iranian diet is devoted to plant foods [12]. Also, bread and rice are recognized as a major source of carbohydrates in Persian diets [14]. In an Asian population like Japan, because of high consumption of rice, the risk of metabolic syndrome and other non-communicable diseases is increasing [15]. Type and quantity of carbohydrates can have some effects on increased risk of cardiovascular disease (CVD) and type 2 diabetes [16–18]. Since the prevalence of obesity and associated co-morbidities in children and adolescents of developing countries is increasing, studies should be conducted regarding dietary pattern and their association with cardio-metabolic risk factors. Accordingly, the current study aimed to evaluate the association of frequency of potato and rice consumption with cardio-metabolic risk factors in Iranian children and adolescents.

## Methods

This multi-centric cross-sectional study was conducted in 2015 as the fifth survey of a surveillance program entitled “Childhood and Adolescence Surveillance and PreventIon of Adult Non- communicable disease” (CASPIAN-V) study. Overall, 14,400 students aged 7–18 years were recruited. They were selected by multistage, stratified cluster sampling from urban and rural areas of 30 provinces of Iran. Overall, 480 students were selected from each province according to the living area (urban/rural) and school grade (primary/ secondary), proportional to size with equal sex ratio. Blood sample was obtained from a sub-sample of 4200 randomly selected students. The study protocol has been described in detail previously [19]. It is summarized here.

### Demographic data

In the current study, two sets of questionnaires were used to collect data from the all students and their parents. Questionnaire from WHO-GSHS was translated into Persian, and then its validity and reliability were assessed [20]. Demographic data including age, sex, residence area, physical activity (PA), and family socio-economic status (SES) were collected by trained health care providers. At least one of the parents was required to be present for answering the questions.

PA was assessed using a valid questionnaire [20]. One week prior to the study, the subjects answered the following two questions: 1) how many days were you physically active for 30 min per day during last week? (responses ranged from 0 to 7 days) 2) How much time per week do you spend in exercise classes at school? (answers ranged from 0 to 3 or more hours).

Screen time (ST) behavior was assessed by asking students questions about how long they spent on watching TV, working with computers, and playing video games. Mean ST of more than 2 h per day was considered high, as sedentary lifestyle [20].

SES was assessed using an approved method provided for the Progress in the International Reading Literacy Study (PIRLS) [19]. Variables like parents’ education and job, type of home (private, rented), type of school (private, governmental), and family asset (e.g., personal computer and private car) were summarized under one main component. Then, SES score was estimated using the principle components analysis (PCA) method [21]. The main component was categorized into three levels. The first, second, and third levels were considered as low, moderate, and high SES, respectively.

### Physical measurements

Anthropometric indices such as body mass index (BMI), wrist circumference (WrC), waist circumference (WC), neck circumference (NC), and hip circumferences (HC) were measured for all students. All measurements were done using a non-elastic tape with standard protocol. Blood pressure (BP) was measured with the students in the arm position using a mercury sphygmanometer with an appropriate cuff size. It was measured twice at 15 min intervals. Systolic and diastolic blood pressure (SBP, DBP) was recorded and the average was recorded [22].

Rice and potato consumption were recorded using non-quantitative food frequency questionnaire (FFQ). In this questionnaire, the students reported the frequency of each food item as daily, weekly, seldom or never. For statistical analysis, the frequency consumption of rice and potato (daily or non-daily) was considered as weekly, seldom or never) consumption. Foods with a high content of sugar, salt, saturated fats and trans fats, and sugar sweetened beverages were considered as low nutrient-dense foods (LNDF).

### Blood sampling

Selected sub-sample students (4200 students out of 14,400 students) accompanying one of their parents were referred to the laboratory for blood sampling. Venous blood samples were collected after 12 h overnight fasting. Centrifugation was done at 2500 to 3000 g for 10 min. Serum samples were stored at − 70 °C and transferred to Isfahan Mahdieh Laboratory by cold chain. Fasting blood glucose (FBS), triglycerides (TG), total cholesterol (TC), low-density lipoprotein cholesterol (LDL-C), and high-density lipoprotein cholesterol (HDL-C) were measured enzymatically using Hita-chi auto- analyzer (Tokyo, Japan) [23, 24].

Metabolic syndrome was defined according to the Adult Treatment Panel III definition as having at least three of the following abnormalities. 1) abdominal obesity (WC < 90th percentile), 2) low HDL-C (HDL < 40 mg/dL except in 15–19-year-old boys, in whom the cut-off was < 45 mg/dL), 3) high TG (TG > 100 mg/ dL), 4) high FBG (FBG > 100 mg/dL), and 5) high BP (SBP or DBP > 90th percentile adjusted by age, sex, and height) [25]. High LDL-C and high TC were defined as LDL-C > 110 mg/dL and TC > 200 mg/dL, respectively [26]. Overweight and obesity were considered as age-sex specific BMI 85th–95th percentile and BMI greater than 95th percentile, respectively [27].

### Statistical analysis

The current study was conducted using a secondary analysis of the CASPIAN-V study. Quantitative variables were expressed as mean (standard deviation) and categorical variables were presented as number (percentage). The Student’s t-test was applied to compare mean difference between the means of quantitative variables. Qualitative variables were assessed using Chi-square test. Association of potato and rice consumption with cardiometabolic risk factors was assessed using linear and logistic regression analysis in different models, consisting of Model I: crude model (without adjustment); Model II: adjusted for age, sex, residence area, SES, PA, ST, LNDF (including sweetened beverages and fast foods consumption), and family history of chronic diseases; Model III: additionally adjusted for BMI only for all cardiometabolic risk factors except anthropometric measures. For all models, in the linear and logistic regression analysis, cardiometabolic risk factors were included as continuous and categorical dependent variable in the model respectively. Results of linear and logistic regression model were reported as beta coefficient with standard error (SE) and odds ratio (OR) with the 95% confidence interval (CI), respectively. The results of the questionnaires were analyzed using clustering analysis to investigate the cluster and design effects. To extrapolate the results to the Iranian children and adolescents, data were weighted directly to the Iranian students according to ministry of education list in 2014, to match the grade (primary/secondary), sex, and living area (rural/urban). All statistical analyses were performed using STATA package version 11.0 (Stata Statistical Software: Release 11. Stata Corp LP. Package. College Station, TX, USA) and a *P*-value< 0.05 was considered statistically significant.

## Results

The participation rates for the whole study and for the blood sampling were 99 and 91.5%, respectively. A total of 14,400 children and adolescents (49.9% girls and 50.4% boys) with mean age of 12.3 ± 3.2 years participated in this study. The frequency of daily consumption of white rice and potato was 84.4 and 21.3%, respectively. Distribution of general characteristics of subjects according to consumption of rice and potato is presented in Supplementary Table 1. Distribution of rice consumption according to living area, level of PA and SES was statistically different (*P* < 0.050). Moreover, association of potato consumption with ST and SES was statistically significant (*P* < 0.050). Table 1 shows the prevalence of cardiometabolic risk factors according to rice and potato consumption. No significant differences were observed between daily and non-daily rice consumers according to prevalence of all cardiometabolic risk factors except high SBP. The prevalence of high SBP in daily potato consumers was significantly higher than non-daily consumers (3.8% vs. 2.9%) (P: 0.01).

**Table 1:** Prevalence of cardio-metabolic risk factors according to rice and potato consumption in Iranian children and adolescents: the CASPIAN-V study

	Rice Consumption			Potato Consumption			Total
	Daily (%)	Nondaily (%)	***P-value***	Daily (%)	Nondaily (%)	***P-value***	
**Abdominal obesity**	2506 (21.0)	460 (21.4)	.677	658 (22.0)	2296 (20.8)	.151	2972 (21.1)
**Over weight**	1131 (9.5)	197 (9.1)	0.630	308 (10.3)	1007 (9.1)	0.053	1330 (9.4)
**Obesity**	1373 (11.5)	240 (11.1)	0.629	342 (11.4)	1268 (11.5)	0.917	1615 (11.4)
**High FBS**	143 (4.3)	18 (3.4)	0.299	41 (4.6)	120 (4.1)	0.467	161 (4.2)
**High TG**	913 (27.6)	151 (28.2)	0.793	237 (26.8)	824 (28.0)	0.489	1065 (27.7)
**High TC**	168 (5.1)	21 (3.9)	0.247	49 (5.5)	140 (4.8)	0.344	189 (4.9)
**Low HDL**	976 (29.5)	157 (29.3)	0.910	261 (29.5)	867 (29.5)	0.970	1134 (29.5)
**High LDL**	569 (17.2)	105 (19.6)	0.180	167 (18.9)	506 (17.2)	0.245	674 (17.5)
**High SBP**	365 (3.1)	71 (3.3)	0.606	114 (3.8)	318 (2.9)	0.010	438 (3.1)
**High DBP**	1214 (10.3)	231 (10.7)	0.521	321 (10.8)	1125 (10.3)	0.435	1450 (10.4)
**High BP**	1350 (11.4)	249 (11.6)	0.848	357 (12.0)	1240 (11.3)	0.324	1604 (11.5)
**MetS**	164 (5.1)	24 (4.6)	0.626	45 (5.2)	143 (5.0)	0.811	188 (5.0)

*MetS* metabolic syndrome, *FBS* fasting blood sugar, *TC* total cholesterol, *TG* triglycerides, *LDL-C* low-density lipoprotein cholesterol, *HDL-C* high-density lipoprotein cholesterol, *BP* blood pressure;

Defined as above the 85th percentile. Overweight: BMI 85th–95th; obesity: BMI >95th; low HDL: 110 mg/dL; high TG: 100 mg/dL; high TC: > 200 mg/dL; high FBS: > 100 mg/dL; high blood pressure: >90th (adjusted by age, sex, height); Abdominal obesity: WHtR> 0.5; MetS, ATP-III criteria;

Table 2 demonstrates the mean (±SD) of anthropometric and cardiometabolic indices according to the rice and potato consumption. The mean values of hip (79.2 ± 14.5) were significantly higher in subjects with daily rice consumption compared to other participants (78.5 ± 14.8) (*P* < 0.050). Diastolic BP was significantly lower in participants with daily rice consumption than non-daily consumption (63.7 ± 10.4 vs. 64.2 ± 10.2), (*P* < 0.001). There was a significant difference in waist to height ratio between participants with daily potato consumption and non-daily potato consumption (0.450 ± 0.06 vs. 0.458 ± 0.06) (*P* < 0.001).

**Table 2:** Mean (±SD) of anthropometric and cardio-metabolic indices according to rice and potato consumption

	Rice Consumption			Potato Consumption		
	Daily Mean (SD)	Nondaily Mean (SD)	***P-value***	Daily Mean (SD)	Nondaily Mean (SD)	***P-value***
**FBS**	91.7 (12.2)	91.0 (11.4)	0.231	91.6 (12.5)	91.6 (12.0)	0.889
**TG**	88.1 (45.9)	87.2 (40.4)	0.663	87.7 (44.9)	88.1 (45.3)	0.827
**TC**	153.6 (27.7)	155.1 (25.1)	0.240	153.9 (28.8)	153.8 (27.0)	0.973
**HDL**	46.2 (9.9)	46.0 (9.9)	0.805	46.4 (10.0)	46.1 (9.9)	0.459
**LDL**	89.9 (22.4)	91.6 (20.5)	0.108	90.2 (23.2)	90.1 (21.8)	0.944
**SBP**	99.0 (13.1)	99.5 (12.8)	0.156	98.9 (13.8)	99.2 (12.8)	0.335
**DBP**	63.7 (10.4)	64.2 (10.2)	0.023	63.6 (10.8)	63.8 (10.3)	0.175
**BMI z- score**	0.01 (0.99)	0.00 (1.03)	0.832	0.02 (1.09)	0.00(0.97)	0.155
**Waist**	66.7 (12.1)	66.4 (12.1)	0.239	67.0 (12.8)	66.6 (11.9)	0.119
**Hip**	79.2 (14.5)	78.5 (14.8)	0.030	79.4 (14.9)	79.0 (14.5)	0.155
**Wrist**	14.7 (1.8)	14.7 (1.9)	0.313	14.7 (2.0)	14.7 (1.8)	0.487
**Neck**	29.8 (3.9)	29.8 (4.1)	0.648	29.8 (4.1)	29.8 (3.9)	0.649
**WHtR**	0.45 (0.06)	0.45 (0.06)	0.121	0.450 (0.06)	0.459 (0.06)	0.011
**WHR**	0.86 (0.19)	0.85 (0.16)	0.09	0.86 (0.17)	0.85 (0.15)	0.42

*FBS* fasting blood sugar, *TC* total cholesterol, *TG* triglycerides, *LDL-C* low-density lipoprotein cholesterol, *HDL-C* high-density lipoprotein cholesterol, *SBP* systolic blood pressure, *DBP* diastolic blood pressure, *WC* Waist circumference, *HC* hip circumference, *NC* neck circumference, *BMI* body mass index, *WHtR* waist to height ratio, *WHR* waist to hip ratio

Association of rice and potato consumption with anthropometric and cardiometabolic indices (as continuous variable) in linear regression analysis is presented in Table 3. Although, in the crude model, daily rice consumer had statistically lower diastolic BP (β: -0.56, SE: 0.24) and HC (β: 0.74, SE: 0.34) compare to nondaily consumers, in the multivariable model theses associations were not statistically significant. In the multivariable linear regression model, daily consumption of potato increased significantly BMI (β: 0.05, SE: 0.2, *p* = 0.010), WC (β: 0.63, SE: 0.24, *p* = 0.008), and HC (β: 0.62, SE: 0.26, *P* = 0.019).

**Table 3:** Association of rice and potato consumption with anthropometric and cardiometabolic indices in linear regression analysis

		Rice (Daily/Nondaily)			Potato (Daily/Nondaily)		
		β	SE	***P-value***	β	SE	***P-value***
**FBS**	Model 1	0.676	0.564	0.231	−.065	.465	.889
	Model 2	0.515	0.642	0.422	.021	.525	.967
	Model 3	0.534	0.646	0.409	.000	.529	.999
**TG**	Model 1	0.918	2.104	0.663	−.380	1.735	.827
	Model 2	0.699	2.308	0.762	−.030	1.887	.987
	Model 3	0.430	2.319	0.853	−.258	1.897	.892
**TC**	Model 1	−1.501	1.277	0.240	.035	1.053	.973
	Model 2	−0.564	1.432	0.694	−.094	1.170	.936
	Model 3	−0.650	1.441	0.652	−.205	1.179	.862
**HDL-C**	Model 1	0.115	0.465	0.805	.283	.382	.459
	Model 2	0.058	0.517	0.911	.449	.422	.288
	Model 3	0.082	0.520	0.875	.432	.425	.309
**LDL-C**	Model 1	−1.661	1.034	0.108	.060	.852	.944
	Model 2	−0.673	1.161	0.562	−.305	.948	.748
	Model 3	−0.731	1.169	0.532	−.353	.955	.712
**SBP**	Model 1	−0.435	0.307	0.156	−.261	.270	.335
	Model 2	−0.295	0.322	0.360	−.182	.279	.515
	Model 3	−0.236	0.317	0.456	−.339	.275	.217
**DBP**	Model 1	−0.557	0.244	0.023	−.293	.216	.175
	Model 2	−0.442	0.266	0.096	−.303	.231	.191
	Model 3	−0.405	0.264	0.125	−.387	.229	.091
**BMI z-score**	Model 1	−0.005	0.023	0.832	.029	.021	.155
	Model 2	−0.016	0.024	0.502	.053	.020	.010
**WC**	Model 1	0.334	0.284	0.239	.390	.250	.119
	Model 2	0.218	0.272	0.423	.628	.236	.008
**HC**	Model 1	0.740	0.341	0.030	.428	.301	.155
	Model 2	0.503	0.305	0.099	.621	.265	.019
**Wrist**	Model 1	−0.045	0.044	0.313	.027	.039	.487
	Model 2	−0.033	0.041	0.419	.031	.036	.385
**NC**	Model 1	0.038	0.093	0.684	.037	.082	.649
	Model 2	−0.035	0.085	0.681	.085	.074	.250
**WHtR**	Model 1	0.002	0.002	0.121	.003	.001	.011
	Model 2	0.001	0.002	0.572	.005	.001	.001
**WHR**	Model 1	−0.008	0.004	0.061	−.003	.004	.455
	Model 2	−0.002	0.004	0.579	−.001	.004	.718

*FBS* fasting blood sugar, *TC* total cholesterol, *TG* triglycerides, *LDL-C* low-density lipoprotein cholesterol, *HDL-C* high-density lipoprotein cholesterol, *SBP* systolic blood pressure, *DBP* diastolic blood pressure, *WC* Waist circumference, *HC* hip circumference, *NC* neck circumference, *BMI* body mass index, *WHtR* waist to height ratio, *WHR* waist to hip ratio

Model 1: Crude Model

Model 2:Adjusted for age, sex, region, SES, PA, ST, family history of chronic diseases, and LNDF (fast foods and sweetened beverages)

Model 3Additionally adjusted for BMI

Association of rice and potato consumption with overweight, obesity and cardiometabolic risk factors in logestic regression analysis is presented in Table 4. In the multivariable model, daily consumption of potato significantly increased odds of overweight (OR: 1.21, 95% CI: 1.04–1.39, *P* = 0.012). Association of potato and rice consumption with other cardiometabolic risk factors was not statistically significant in the multivariable model (*p*-value> 0.05).

**Table 4:** Association between rice and potato consumption with cardio-metabolic risk factors in logistic regression analysis

		Rice Consumption			Potato Consumption		
		OR	95% CI	p-Value	OR	95% CI	p-Value
**High FBS**	Model 1	1.301	.790–2.143	.301	1.144	.796–1.645	.467
	Model 2	1.123	.665–1.898	.664	1.332	.906–1.958	.144
	Model 3	1.110	.657–1.877	.697	1.308	.888–1.927	.174
**High TG**	Model 1	.973	.794–1.192	.793	.942	.795–1.116	.489
	Model 2	.947	.756–1.185	.633	.935	.775–1.127	.479
	Model 3	.942	. 752–1.181	.607	.922	.764–1.113	.398
**High TC**	Model 1	1.313	.827–2.087	.249	1.175	.841–1.641	.345
	Model 2	1.433	.841–2.440	.185	1.206	.835–1.741	.318
	Model 3	1.418	.832–2.417	.199	1.187	.820–1.720	.364
**Low HDL**	Model 1	1.012	.828–1.236	.910	1.003	.851–1.183	.970
	Model 2	1.053	.840–1.321	.653	.955	.794–1.150	.630
	Model 3	1.055	.840–1.325	.645	.948	.786–1.142	.571
**High LDL**	Model 1	.854	.677–1.076	.181	1.122	.924–1.362	.245
	Model 2	.896	.689–1.164	.409	1.152	.930–1.426	.195
	Model 3	.890	.685–1.157	.383	1.152	.930–1.427	.196
**High BP**	Model 1	.986	.854–1.138	.848	1.065	.940–1.207	.324
	Model 2	1.019	.865–1.201	.820	1.063	.925–1.223	.389
	Model 3	1.034	.875–1.221	.697	1.048	.910–1.206	.518
**Mets**	Model 1	1.115	.719–1.730	.626	1.043	.739–1.471	.811
	Model 2	1.087	.668–1.768	.738	1.079	.738–1.577	.694
	Model 3	1.104	.667–1.829	.700	1.014	.685–1.501	.944
**Abdominal obesity**	Model 1	.976	.873–1.092	.677	1.074	.974–1.185	.151
	Model 2	.942	.830–1.069	.357	1.114	.999–1.242	.052
**Overweight**	Model 1	1.040	.89–1.22	.631	1.142	.99–1.30	.053
	Model 2	1.029	.860–1.230	.757	1.207	1.042–1.399	.012
**Obesity**	Model 1	1.037	.896–1.199	.629	.993	.875–1.128	.917
	Model 2	.981	.831–1.158	.820	1.098	.956–1.262	.186

*MetS* metabolic syndrome, *FBS* fasting blood sugar, *TC* total cholesterol, *TG* triglycerides, *LDL-C* low-density lipoprotein cholesterol, *HDL-C* high-density lipoprotein cholesterol, *BP* blood pressure;

Defined as above the 85th percentile. Overweight: BMI 85th–95th; obesity: BMI >95th; low HDL: 110 mg/dL; high TG: 100 mg/dL; high TC: > 200 mg/dL; high FBS: > 100 mg/dL; high blood pressure: >90th (adjusted by age, sex, height); Abdominal obesity: WHtR> 0.5; MetS, ATP-III criteria;

Model 1: Crude Model

Model 2: Adjusted for age, sex, region, SES, PA, ST, family history of chronic diseases, and food habits (fast foods and sweetened beverages)

Model 3: Additionally adjusted for BMI

## Discussion

In this national-wide study, we found significant associations of potato consumption with some cardio-metabolic risk factors. In potato consumers and after adjustment for potential confounders, some risk factors such as BMI, WC, and HC remained significant. The results also revealed that in the multivariable model, daily rice consumption was not associated with cardiometabolic risk factors. Our inability to identity positive associations between rice consumption and alteration of other cardio-metabolic risk factors may be related to change in eating habits of the studied population when body composition such as overweight and obesity is altered. Also, some specific difficulties in evaluating food consumption of children and adolescents such as limitation of the selected dietary survey, underreporting, etc. should be considered as the possible reasons for the mentioned issue.

Numerous studies have revealed that diet composition may play an essential role in development of obesity and its risk factors in adults [28, 29]. Data regarding the effects of dietary components on development of childhood obesity and related risk factors have so far been inconclusive.

There are some inconsistencies in the studies assessing the relationship between refined grains and CVD risk factors. Some of them found no association [30] and others demonstrated even an increment [31]. Some studies have reported positive associations between white rice consumption and CVD risk factors. According to some reports, higher intake of refined grains is associated with higher risk of type II diabetes [32], stroke [33], and hypertriglyceridemia [34]. However, other studies have found little association [16], with the potential exception of an association between white rice and type 2 diabetes [17]. Indian researchers found that rice, as a major source of carbohydrate, in comparison with other sources of carbohydrates, had a weaker effect on rising TG level [35]. The results of a clinical trial conducted on patients with coronary artery disease showed that after replacing refined rice with legume powder and whole grain products, as a source of carbohydrates, glucose and insulin concentrations changed significantly [36]. Other studies showed that refined grains had no effects on risk of hypertension [37], heart diseases, and diabetes type II [31, 38]. Also, no significant association was observed between refined grains’ intake and concentration of fasting insulin in Framingham Cohort Study [39].

The current study revealed that in subjects who consumed potato on a daily basis, the possibility of overweight increased. Inducing a faster rise in glucose level and insulin response maybe one of the possible mechanisms involved in the observed association. Since energy density is considered as a major factor contributing to overconsumption, explaining the relationship between specific foods consumption and excess weight gain is complex.

To enhance flavor in potatoes, they are mostly prepared by oils and fats. So, nutritional value of potatoes and potato products might be overlooked [30]. Due to confounders such as cooking method and storage, the association between dietary potato intake and health is complex [40]. Despite recent recommendations to consume starchy foods, we should consider the above-mentioned points for daily consumers of potato even in local foods [41].

Mozaffarian et al. evaluated the intake of 13 groups of foods and beverages among adults for 4 years. They found that weight gain was significantly higher in subjects who increased their daily intake of potato chips, potato, sugar-sweetened beverages, and processed meat, as compared with those who increased daily intake of vegetables, whole grains, fruit, yogurt, and nuts [42].

In a study conducted by Dong et al. on 7–13-year-old children, they found that high protein and fiber foods (dietary components that increase satiety and induce lower glycemic index) are negatively associated with excess weight gain [43].

In the present study, subjects with daily consumption of rice had lower DBP compared to non-daily consumers. The results from recent randomized controlled trials are inconsistent with our findings. They demonstrated that low-carbohydrate diets can decrease systolic and diastolic blood pressure [44, 45]. Weight loss would be the driving factor in decreasing blood pressure levels [46]. The observed association in the current study might be related to cross-sectional design of the study. Also, BP results were averages from only two consequent measurements. This method is not a precise methodology for measuring BP; it might be useful to recognize hypertensive subjects [47].

Our study revealed that daily fast foods and daily rice consumers had higher SES levels which are basically because of the higher price of such food items in Iran. While, daily potato consumers had lower SES because potato is very cheap food item in all parts of Iran. Also, current behavior regarding consumption of sweetened beverages and the possible predictors should be examined by using theory of planed behavior (TPB) in the future.

This study has some specific limitations. First, as a result of cross-sectional nature of the study, the obtained associations should be considered with caution. Second, data of portion size, quantification information, and type of consumed potato and rice were not collected in our study. Third, we were not able to estimate energy intake in the current study. Fourth, measurement errors are inevitable due to difficulties in evaluating food consumption of children and adolescents. Also, recall bias might be happen because of using self-reported physical activity questioner. The strengths of this study are its large sample size and novelty in the pediatric age group.

## Conclusions

Daily consumption of potato was significantly associated with higher anthropometric measures whereas rice consumption had no statistically significant association with cardiometabolic risk factors. The prevalence of SBP was significantly higher in daily potato consumers compared to others. Therefore, it is recommended that future research examines the possible obesogenic effects of intake of potato on children and adolescents and also, we should consider this point that the evidence of single foods needs to be considered in the context of the total diet.

Despite numerous difficulties in assessing dietary habits of children and adolescents, such studies should be considered a main basis for devising interventions at an early age.

## Supplementary information

**Additional file 1: Supplementary Table 1:** General characteristics and some food habits of subjects according to rice and potato consumption in Iranian children and adolescents: the CASPIAN-V study.

**Additional file 2.**

## Data Availability

The dataset supporting the conclusions of this article will not be shared publicly, to ensure participants’ privacy.

## Abbreviations

Mets: Metabolic syndrome
CVD: Cardiovascular disease
CASPIAN-V: Childhood and adolescence surveillance and PreventIon of adult non- communicable disease
PA: Physical activity
SES: Family socio-economic status
ST: Screen time
PIRLS: Progress in the international Reading literacy study
PCA: Principle components analysis
BMI: Body mass index
WrC: Wrist circumference
WC: Waist circumference
NC: Neck circumference
HC: Hip circumferences
BP: Blood pressure
SBP: Systolic blood pressure
DBP: Diastolic blood pressure
FFQ: Food frequency questionnaire
LNDF: Low nutrient-dense foods
FBS: Fasting blood glucose
TG: Triglycerides
TC: Total cholesterol
LDL-C: Low-density lipoprotein cholesterol
HDL-C: High-density lipoprotein cholesterol
SE: Standard error
OR: Odds ratio
CI: Confidence interval
TPB: Theory of planed behavior
