# Supplementary material for: Is frequency of potato and white rice consumption associated with cardiometabolic risk factors in children and adolescents: the CASPIAN-V study
Source: BMC Cardiovasc Disord. 2020 May 19;20:239. doi: 10.1186/s12872-020-01524-y (PMC7236272; doi:10.1186/s12872-020-01524-y)
Supplement: Supplementary file 1 — Additional file 1: Supplementary Table 1: General characteristics and some food habits of subjects according to rice and potato consumption in Iranian children and adolescents: the CASPIAN-V study. [file 12872_2020_1524_MOESM1_ESM.docx]

**Supplementary table 1:** General characteristics and some food habits of subjects according to rice and potato consumption in Iranian children and adolescents: the CASPIAN-V study

|  | | Rice Consumption | | | Potato Consumption | | |
| --- | --- | --- | --- | --- | --- | --- | --- |
|  |  | Daily | Nondaily | *P-value* | Daily | Nondaily | *P-value* |
| Age (year) ^1^ | | 12.2(3.1) | 12.3(3.1) | 0.24 | 12.3(3.2) | 12.3(3.2) | 0.94 |
| Sex^2^ | Boy | 5944 (84.6) | 1081(15.4) | 0.96 | 1524 (21.2) | 5664 (78.8) | 0.50 |
|  | Girl | 6106 (84.6) | 1108 (15.4) |  | 1516 (21.7) | 5484 (78.3) |  |
| Living area ^2^ | Urban | 8676(85.4) | 1488(14.6) | <0.001 | 2133(21.1) | 7997 (78.9) | 0.08 |
|  | Rural | 3374(82.8) | 701 (17.2) |  | 907(22.4) | 3151 (77.6) |  |
| ST^2^ | Low | 9820 (84.5) | 1801 (15.5) | 0.9 | 2407 (20.8) | 9164 (79.2) | <0.001 |
|  | High | 1890 (84.6) | 344 (15.4) |  | 560 (25.1) | 1671 (74.9) |  |
| PA ^2^ | Low | 4901 (83.1) | 997 (16.9) | <0.001 | 1304 (22.2) | 4576 (77.8) | 0.08 |
|  | High | 7072 (86.2) | 1136 (13.8) |  | 1714 (21) | 6461 (79) |  |
| SES^2^ | Low | 3695 (81.0) | 865 (19.0) | <0.001 | 1037 (22.9) | 3497 (77.1) | 0.001 |
|  | Moderate | 3871 (85.8) | 641 (14.2) |  | 884 (19.7) | 3608 (80.3) |  |
|  | High | 3991 (87.7) | 560 (12.3) |  | 984 (21.6) | 3562 (78.2) |  |
| Sweetened beverages consumption | Daily | 1568(82.8) | 326(17.2) | 0.02 | 331(17.4) | 1566(82.6) | <0.001 |
|  | Non-daily | 10314(84.8) | 1844 (15.2) |  | 2620(21.6) | 9484 (78.4) |  |
| Fast food consumption | Daily | 1432 (87.8) | 206(12.6) | 0.001 | 1303 (79.5) | 335 (20.5) | 0.32 |
|  | Non-daily | 10583 (84.3) | 1977 (15.7) |  | 9822 (78.5) | 2693 (21.5) |  |
| ST: screen time, PA: physical activity; SES: socioeconomic status  ^1^ are presented as mean (SD)  ^2^ are presented as number (%) | | | | | | | |
