# Supplementary material for: Is frequency of potato and white rice consumption associated with cardiometabolic risk factors in children and adolescents: the CASPIAN-V study
Source: BMC Cardiovasc Disord. 2020 May 19;20:239. doi: 10.1186/s12872-020-01524-y (PMC7236272; doi:10.1186/s12872-020-01524-y)
Supplement: Supplementary file 2 — Additional file 2. [file 12872_2020_1524_MOESM2_ESM.docx]

**A:** **Information on nutrition**

| **Question** | **Answer** | **Code** |
| --- | --- | --- |
| What time do you usually have your breakfast? | **18. School days** |  |
|  | **19. Holidays** (Fridays and other official holidays): |  |
| How many days a week do you often have breakfast? (We mean having more than a glass of milk or a cup of tea). Please answer for both holidays and school days. | **20. School days** |  |
|  | 🞏1.Never 🞏2.One day 🞏3. Two days  🞏4.Three days 🞏5. Four days 🞏6.Five days 🞏7. Six days |  |
|  | **21. Holidays** (Fridays and other official holidays) |  |
|  | 🞏1.I don’t usually have breakfast 🞏2. I Usually have  breakfast |  |
| What time do you usually have your lunch? | **22. School days** |  |
|  | **23. Holidays** (Fridays and other official holidays): |  |
| How many days a week do you have your lunch at noon? (lunch is more than just a glass of juice or some both biscuits). Please answer for holidays and school days. | **24. School days** |  |
|  | 🞏1.Never 🞏2. One day 🞏3. Two days  🞏4. Three days 🞏5. Four days 🞏6. Five days 🞏7. Six days |  |
|  | **25. Holidays** (Fridays and other official holidays) |  |
|  | 🞏1.I don’t usually have lunch 🞏2. I usually have  lunch |  |
| What time do you usually have your dinner? | 26. School days: … |  |
|  | 27. Holidays (Fridays and other official holidays): |  |
| How many nights a week do you have dinner? (We mean having more than a glass of juice or some biscuits) please answer for both Fridays and school days. | 28. School days |  |
|  | 1.Never 2.one evening 3.two evening  3.Three evening 5.four evening 6.five evening 7.six evening |  |
|  | 29. Holidays (Fridays and other official holidays) |  |
|  | 1.I don’t usually have dinner  2 I usually have dinner. |  |

|  | **Question** | **Answer** | **Code** |
| --- | --- | --- | --- |
| How many times do you use each of the following food groups? | | |  |
| 30 | Cakes, cookies, sweets, biscuits, chocolate | 🞏1.Daily 🞏2.Weekly 🞏3.Seldom 🞏4.Never |  |
| 31 | Snacks, chips, pretzels | 🞏1.Daily 🞏2.Weekly 🞏3.Seldom 🞏4.Never |  |
| 32 | Ordinary soft drinks | 🞏1.Daily 🞏2.Weekly 🞏3.Seldom 🞏4.Never |  |
| 33 | Diet sodas | 🞏1.Daily 🞏2.Weekly 🞏3.Seldom 🞏4.Never |  |
| 34 | Non-alcoholic beer | 🞏1.Daily 🞏2.Weekly 🞏3.Seldom 🞏4.Never |  |
| 35 | Fresh fruits, dried fruits (sheet peaches,  dried figs, raisins, etc.) | 🞏1.Daily 🞏2.Weekly 🞏3.Seldom 🞏4.Never |  |
| 36 | Fresh fruit juice | 🞏1.Daily 🞏2.Weekly 🞏3.Seldom 🞏4.Never |  |
| 37 | Canned Juices | 🞏1.Daily 🞏2.Weekly 🞏3.Seldom 🞏4.Never |  |
| 38 | Fresh or cooked vegetables (vegetables,  (carrots, salad | 🞏1.Daily 🞏2.Weekly 🞏3.Seldom 🞏4.Never |  |
| 39 | Potato | 🞏1.Daily 🞏2.Weekly 🞏3.Seldom 🞏4.Never |  |
| 40 | Rice | 🞏1.Daily 🞏2.Weekly 🞏3.Seldom 🞏4.Never |  |
| 39 | Milk | 🞏1.Daily 🞏2.Weekly 🞏3.Seldom 🞏4.Never |  |
| 40 | Yogurt | 🞏1.Daily 🞏2.Weekly 🞏3.Seldom 🞏4.Never |  |
| 41 | Cheese | 🞏1.Daily 🞏2.Weekly 🞏3.Seldom 🞏4.Never |  |
| 42 | Sausage and salami, pizza, burgers | 🞏1.Daily 🞏2.Weekly 🞏3.Seldom 🞏4.Never |  |
| 43-1 | Tea | 🞏1.Daily 🞏2.Weekly 🞏3.Seldom 🞏4.Never |  |
| 43-2 | How many cups of tea do you drink a day? | 🞏1.None 🞏2.One to two 🞏3.Three to four 🞏4.More than four |  |

**B:** **Physical activity**

|  | **Question** | **Answer** | | | **Code** |
| --- | --- | --- | --- | --- | --- |
| 52 | How many days last week did you have physical activity for a total of 30 minutes (half an hour) a day? | 🞏1.Never 🞏2.One day  🞏3.Two days 🞏4.Three days  🞏5.Four days 🞏6.Five days  🞏7.Six days 🞏7.Seven days | | |  |
| 53 | Are physical education classes held regularly  at your school? | 🞏1.Yes 🞏2.No | | |  |
| 54 | How many hours a week do you attend  physical education classes regularly at school? | 🞏1.Nil  🞏3.Two hours | 🞏2.One hour  🞏4.Three hours or | more |  |
| 55 | How do you usually go to school? | 🞏1.School bus  🞏4.Car  🞏5.Bicycle | 🞏2.Public bus  🞏3.On foot | |  |

**C: Leisure activities**

| **Question** | **Answer** | **Code** |
| --- | --- | --- |
| How many hours a day do you watch TV (or video) in your free time?  Please answer for both Fridays and holidays and school days. | **56. School days** |  |
|  | 🞏1.Nil 🞏2.About one hour 🞏3.About two hours  🞏4.About three hours 🞏5.About four hours or more |  |
|  | **57. Holidays** (Fridays and other official holidays) |  |
|  | 🞏1.Nil 🞏2.About one hour 🞏3.About two hours  🞏4.About three hours 🞏5.Four hours or more |  |
| How many hours a day do you spend on doing homework? (Please answer for both Fridays and holidays and school days) | **58. School days** |  |
|  | 🞏1.Nil 🞏2.About one hour 🞏3.About two hours  🞏4.About three hours 🞏5.About four hours or more |  |
|  | **59. Holidays** (Fridays and other official holidays) |  |
|  | 🞏1.Tenth 🞏2.About one hour 🞏3.About two hours  🞏4.About three hours 🞏5.About four hours or more |  |
| How many hours a day do you work with computer (playing, sending emails, chatting or searching on the internet)? | **60. School days** |  |
|  | 🞏1.Nil 🞏2.About one hour 🞏3.About two hours  🞏4.About three hours 🞏5.Four hours or more |  |
|  | **61. Holidays (Fridays and other official holidays)** |  |
|  | 🞏1.Nil 🞏2.About one hour 🞏3.About two hours  🞏4.About three hours 🞏5.Four hours or more |  |
| How many hours do you usually sleep per day? (Total Day and Night) | **62. School days** |  |
|  | Hours beginning time of sleep: …. |  |
|  | **63. Holidays** (Fridays and other official holidays) |  |
|  | Hours beginning time of sleep: …. |  |

**D: Clinical examinations findings**

|  | **Clinical examinations** | | **Code** |
| --- | --- | --- | --- |
| 128 | Weight (with minimal clothing and no shoes) | □□□.□ Kg |  |
| 129 | Height (standing position without  shoes) | □□□.□ cm |  |
| 130 | Waist circumference of (standing  position) | □□□.□ cm |  |
| 131 | Wrist circumference (dominant hand) | □□□.□ cm |  |
| 132 | Hip circumference | □□□.□ cm |  |
| 133 | Neck circumference (standing position) | □□□.□ cm |  |
| 134 | Systolic blood pressure | ……….. mmHg |  |
| 135 | Diastolic blood pressure | ……….. mmHg |  |
